# Impactful Recommendations for Older Veterans: Experiences of Neuropsychologists Using Telehealth

**DOI:** 10.1093/geroni/igaf122.2551

**Published:** 2025-12-31

**Authors:** D J King, Maureen O’Connor, Malissa Kraft

**Affiliations:** Bedford VA Medical Center, Bedford, Massachusetts, United States; Bedford Veterans Health Care System, Bedford, Massachusetts, United States; Bedford VA Medical Center, Bedford, Massachusetts, United States

## Abstract

Access to in-person healthcare is a barrier for older adult Veterans who live in rural areas or cannot access reliable transportation. This is especially concerning for Veterans with cognitive or neurodegenerative concerns, for which a neuropsychological evaluation is essential for diagnosis and treatment. In response, Veteran Affairs (VA) offers synchronous telehealth services through VA Video Connect (VVC) to Veterans in their homes or through Clinical Video Telehealth (CVT) at nearby Community-Based Outpatient Clinics (CBOCs). Five neuropsychologists were interviewed about their experiences conducting neuropsychological evaluations via telehealth and their challenges offering subsequent recommendations. Of the five providers, three conducted tele-neuropsychological evaluations full-time and two split their time between in-person and telehealth care. Two providers were working with a national Veteran sample, two providers with a regional sample, and one provider with a local sample, each seeing two to four cases per week on average. Reported challenges when conducting tele-neuropsychological evaluations included technological illiteracy, Veteran discomfort, distractors and background noise, differences across VA facilities, scheduling complications, and Veteran transportation issues. Common trends emerged regarding challenges to making virtual recommendations for Veterans with barriers to in-person care, including uncertainty about available VA services for remotely seen Veterans, limitations in accessing community resources, limitations in transportation services for in-person care recommendations, and variable assistance from referring providers. We will also present our manualized recommendations guide for tele-neuropsychologists, as well as preliminary feedback about the guide. Though developed for tele-neuropsychologists, the guide has potential wide scale usability for all providers working with older Veterans.

